# Dr. Charles Henry Brown

**Published:** 1901-11

**Authors:** 


Dr. Charles Henry Brown, of New York, died October 15, 1901, aged 45 years. He was for many years managing editor of the *Journal of Nervous and Mental Diseases*. Dr. Brown came from a family of doctors and was himself a noted physician. He is survived by a widow and two daughters.

